# Semi-Supervised Learning of Statistical Models for Natural Language Understanding

**DOI:** 10.1155/2014/121650

**Published:** 2014-07-20

**Authors:** Deyu Zhou, Yulan He

**Affiliations:** ^1^School of Computer Science and Engineering, Key Laboratory of Computer Network and Information Integration, Ministry of Education, Southeast University, Nanjing 210096, China; ^2^School of Engineering and Applied Science, Aston University, Birmingham B4 7ET, UK

## Abstract

Natural language understanding is to specify a computational model that maps sentences to their semantic mean representation. In this paper, we propose a novel framework to train the statistical models without using expensive fully annotated data. In particular, the input of our framework is a set of sentences labeled with abstract semantic annotations. These annotations encode the underlying embedded semantic structural relations without explicit word/semantic tag alignment. The proposed framework can automatically induce derivation rules that map sentences to their semantic meaning representations. The learning framework is applied on two statistical models, the conditional random fields (CRFs) and the hidden Markov support vector machines (HM-SVMs). Our experimental results on the DARPA communicator data show that both CRFs and HM-SVMs outperform the baseline approach, previously proposed hidden vector state (HVS) model which is also trained on abstract semantic annotations. In addition, the proposed framework shows superior performance than two other baseline approaches, a hybrid framework combining HVS and HM-SVMs and discriminative training of HVS, with a relative error reduction rate of about 25% and 15% being achieved in *F*-measure.

## 1. Introduction

Given a sentence such as “I want to fly from Denver to Chicago,” its semantic meaning can be represented as FROMLOC(CITY(Denver)) TOLOC(CITY(Chicago)).

Natural language understanding can be considered as a mapping problem where the aim is to map a sentence to its semantic meaning representation (or abstract semantic annotation) as shown above. It is a* structured classification* task which predicts output labels (semantic tag or concept sequences) from input sentences where the output labels have rich internal structures.

Early approaches rely on hand-crafted semantic grammar rules to fill slots in semantic frames using word pattern and semantic tokens [1, 2]. Such rule-based approaches are typically domain-specific and often fragile. In contrast, statistical approaches are able to accommodate the variations found in real data and hence can in principle be more robust. They can be categorized into three types: generative approaches, discriminative approaches, and a hybrid of the two.

Generative approaches learn the joint probability model, *P*(*C*, *S*), of input sentence *S* and its semantic tag sequence *C*, then compute *P*(*C*∣*S*) using Bayes' rule, and finally take the most probable semantic tag sequence *C*. The hidden Markov model (HMM), a generative model, has been predominantly employed in statistical semantic parsing. It models sequential dependencies by treating a semantic parse sequence as a Markov chain, which leads to an efficient dynamic programming formulation for inference and learning. Discriminative approaches directly model posterior probability *P*(*C*∣*S*) and learn mappings from *S* to *C*. Conditional random fields (CRFs), as one representative example, define a conditional probability distribution over label sequence given an observation sequence, rather than a joint distribution over both label and observation sequences [3]. Another example is the hidden Markov support vector machines (HM-SVMs) [4] which combine the flexibility of kernel methods with the idea of HMMs to predict a label sequence given an input sequence.

Nevertheless, statistical models mentioned above require fully annotated corpora for training which are difficult to obtain in practical applications. It thus motivates the investigation of train statistical models on abstract semantic annotations without the use of expensive token-style annotations. This is a highly challenging problem because the derivation from each sentence to its abstract semantic annotation is not annotated in the training data and is considered hidden.

A hierarchical hidden state structure could be used to model embedded structural context in sentences, such as the hidden vector state (HVS) model [5], which learns a probabilistic pushdown automaton. However, it cannot incorporate a large number of correlated lexical or syntactic features in input sentences and cannot handle any arbitrary embedded relations since it only supports right-branching semantic structures.

In this paper, we propose a novel learning framework to train statistical models from unaligned data. Firstly, it generates semantic parses by computing expectations using initial model parameters. Secondly, parsing results are then filtered based on a measure describing the level of agreement with the sentence abstract semantic annotations. Thirdly, the filtered parsing results are fed into model learning. With the reestimated parameters, the learning of statistical models goes to the next iteration until no more improvements could be achieved. The proposed framework has two advantages: one is that only abstract semantic annotations are required for training without the explicit word/semantic tag alignment; and another is that the proposed learning framework can be easily extended for training any discriminative models on abstract semantic annotations.

We apply the proposed learning framework on two statistical models, CRFs and HM-SVMs. Experimental results on the DARPA communicator data show that the framework on both CRFs and HM-SVMs outperforms the baseline approach, the previously proposed HVS model. In addition, the proposed framework shows superior performance than two other approaches, a hybrid framework combining HVS and HM-SVMs and discriminative training of HVS, with a relative error reduction rate of about 25% and 15% being achieved in *F*-measure.

The rest of this paper is organized as follows.  Section 2 gives a brief introduction of CRFs and HM-SVMs, followed by a review on the existing approaches for training semantic parsers on abstract annotations. The proposed framework is presented in Section 3. Experimental setup and results are discussed in Section 4. Finally, Section 5 concludes the paper.

## 2. Related Work

In this section, we first briefly introduce CRFs and HM-SVMs. Then, we review the existing approaches for training semantic parsers on abstract semantic annotations.

### 2.1. Statistical Models

Given a set of training data *D* = {(*S*_*i*_, *C*_*i*_), *i* = 1,…, *N*}, to learn a function that assigns to a sequence of words *S* = {*s* ^1^, *s* ^2^,…, *s* ^*T*^}, *s* ^*i*^ ∈ **s**, *i* = 1,…, *T*, a sequence of semantic concepts or tags *C* = {*c* ^1^, *c* ^2^,…, *c* ^*T*^}, *c* ^*i*^ ∈ **c**, *i* = 1,…, *T*, a common approach is to find a discriminant function *F* : *S* × *C* → *R* that assigns a score to every input *S* ∈ *S* and every semantic tag sequence *C* ∈ *C*. In order to obtain a prediction *f*(*S*) ∈ *C*, the function is maximized with respect to *f*(*S*) = arg max⁡_*C*∈*C*_⁡*F*(*S*, *C*).

#### 2.1.1. Conditional Random Fields (CRFs)

Linear-chain CRFs, as a discriminative probabilistic model over sequences of feature vectors and label sequences, have been widely used to model sequential data. This model is analogous to maximum entropy models for structured outputs. By making a first-order Markov assumption on states, a linear-chain CRF defines a distribution over state sequence *C* = {*c* ^1^, *c* ^2^,…, *c* ^*T*^} given an input sequence *S* = {*s* ^1^, *s* ^2^,…, *s* ^*T*^} (*T* is the length of the sequence) as

(1) $$\begin{array}{c} p\left(C\;|\;S\right)=\frac{\Pi_{t}\Phi_{t}\left(c^{t-1},c^{t},S\right)}{Z\left(S\right)}, \end{array}$$

where the partition function *Z*(*S*) is the normalization constant that makes the probability of all state sequences sum to one and is defined as *Z*(*S*) = Σ_*c*_Π_*t*_Φ_*t*_(*c* ^*t*−1^, *c* ^*t*^, *S*).

By exploiting the Markov assumption, *Z*(*S*) can be calculated efficiently by variants of the standard dynamic programming algorithms used in HMM instead of summing over the exponentially many possible state sequences *c*. Φ(*c* ^*t*−1^, *c* ^*t*^, *S*) can be factorized as

(2) $$\begin{array}{c} \Phi \left(c^{t-1},c^{t},S\right)=\exp\left(\Sigma_{k}\theta_{k}f_{k}\left(c^{t-1},c^{t},S,t\right)\right), \end{array}$$

where *θ*_*k*_ is the real weight for each feature function *f*_*k*_(*c* ^*t*−1^, *c* ^*t*^, *S*, *t*). The feature functions describe some aspect of a transition from *c* ^*t*−1^ to *c* ^*t*^ as well as *c* ^*t*^ and the global characteristics of *S*. For example, *f*_*k*_ may have value 1 when POS(*s* ^*t*−1^) = DT and POS(*s* ^*t*^) = NN, which means that the previous word *s* ^*t*−1^ has the POS tag “DT” (determiner) and the current word *s* ^*t*^ has the POS tag “NN” (noun, singular common). The final model parameters for CRFs are a set of real weights Θ = {*θ*_*k*_}, one for each feature.

#### 2.1.2. Hidden Markov Support Vector Machines (HM-SVMs)

For HM-SVMs [4], the function *F*(*S*, *C*) is assumed to be linear in some combined feature representation of *S* and *C*; *F*(*S*, *C*): = 〈*w*, Φ(*S*, *C*)〉. The parameters *w* are adjusted so that the true semantic tag sequence *C*_*i*_ scores higher than all other tag sequences *C* ∈ *C*_*i*_ : = *C*∖*C*_*i*_ with a large margin. To achieve the goal, the following optimization problem is solved:

(3)  min⁡ξi∈R,w∈F Cons∑iξi+12||w||2 s.t.  〈w,Φ(S,Ci)〉−〈w,Φ(S,C)〉≥1−ξi,∀i=1,…N, C∈C∖Ci,

where *ξ*_*i*_ is nonnegative slack variables allowing one to increase the global margin by paying a local penalty on some outlying examples and Cons dictates the desired tradeoff between margin size and outliers. To solve (3), the dual of the equation is solved instead. The solution $\hat{w}$ can be written as

(4) $$\begin{array}{c} \hat{w}=\overset{N}{\underset{i=1\;\;}{\sum }}\underset{C\in C}{\sum }\alpha_{i}\left(C\right)\Phi \left(S_{i},C\right), \end{array}$$

where *α*_*i*_(*C*) is the Lagrange multiplier of the constraint associated with example *i* and *C*_*i*_.

### 2.2. Training Statistical Models from Lightly Annotated Data

Semantic parsing can be viewed as a pattern recognition problem and statistical decoding can be used to find the most likely semantic representation. The majority of statistical approaches to semantic parsing rely on fully annotated corpora. There have been some prior works on learning semantic parsers that map natural language sentences into a formal meaning representation such as first-order logic [6–10]. However these systems either require a hand-built, ambiguous combinatory categorical grammar template to learn a probabilistic semantic parser [11] or assume the existence of an unambiguous, context-free grammar of the target meaning representations [6, 7, 9, 12, 13]. Furthermore, they have only been studied in two relatively simple tasks, GeoQuery [14] for US geography query and RoboCup (http://www.robocup.org/) where coaching instructions are given to soccer agents in a simulated soccer field.

He and Young [5] proposed the hidden vector state (HVS) model based on the hypothesis that a suitably constrained hierarchical model may be trainable without treebank data whilst simultaneously retaining sufficient ability to capture the hierarchical structure needs to robustly extract task domain semantics. Such a constrained hierarchical model can be conveniently implemented using the HVS model which extends the* flat-concept* HMM model by expanding each state to encode the stack of a pushdown automaton. This allows the model to efficiently encode hierarchical context, but because stack operations are highly constrained it avoids the tractability issues associated with full context-free stochastic models such as the hierarchical HMM. Such a model is trainable using only lightly annotated data and it offers considerable performance gains compared to the flat-concept model.

Conditional random fields (CRFs) have been extensively studied for sequence labeling. Most applications require the availability of fully annotated data, that is, an explicit alignment of sentence and word-level labels. There have been some attempts to train CRFs from a small set of labeled data and a large set of unlabeled data. In these approaches, a training objective is redefined to combine the conditional likelihood of labeled data and unlabeled data. Jiao et al. [15] extended the minimum entropy regularization framework to the structured prediction case so a training objective that combines unlabeled conditional entropy with labeled conditional likelihood is yielded. Mann and McCallum [16] augmented the traditional conditional likelihood objective function with an additional term that aims to minimize the predicted label entropy on unlabeled data. Entropy regularization was employed for semisupervised learning. In [17], a training objective combining the conditional likelihood on labeled data and the mutual information on unlabeled data is proposed. It is based on the rate distortion theory in information theory. Mann and Mccallum [18] used labeled features instead of fully labeled instances to train linear-chain CRFs. Generalized expectation criteria are used to express a preference for parameter settings in which the model distribution on unlabeled data matches a target distribution. They tested their approach on the classified advertisements data set (Classified) [19] consisting of classified advertisements for apartment rentals in the San Francisco Bay Area with 12 fields being labeled for each of the advertisements, including size, rent, neighborhood, and features. With only labeled features, their approach gave a mediocre result with 68.3% accuracy being achieved. With an additional inclusion of 100 labeled instances, the accuracy is increased to 80%. The DARPA communicator data used in our experiment appear to be more complex than the Classified data since semantic annotations in the DARPA communicator data describe embedded structural context in sentences while semantic labels in the Classified data do not represent any hierarchical relations.

## 3. The Proposed Framework

Given the training data *D* = {(*S*_1_, *A*_1_),…, (*S*_*N*_, *A*_*N*_)}, where *A*_*i*_ is the abstract annotation for sentence *S*_*i*_, the parameters Θ will be estimated through a maximum likelihood procedure. The log-likelihood of *L*(Θ) with expectation over the abstract annotation is calculated as follows:

(5) $$\begin{array}{c} L\left(\Theta \right)=\overset{N}{\underset{i}{\sum }}\underset{C_{i}^{u}}{\sum }P\left(C_{i}^{u}\;|\;S_{i}\right)\log P\left(C_{i}^{u}\;|\;S_{i}\right), \end{array}$$

where *C*_*i*_^*u*^ is the unknown semantic tag sequence of the *i*th word sequence. To learn statistical models, we extended the use of expectation maximization (EM) algorithm to estimate model parameters. The EM algorithm [20] is widely employed in statistical models for parameter estimation when the model depends on unobserved latent variables. Given a set of observed data *D*, a set of unobserved latent data, or missing values *D* ^*u*^, the EM algorithm seeks to find the maximum likelihood estimation of the marginal likelihood

(6) $$\begin{array}{c} L\left(D\;|\;\theta \right)=\underset{D^{u}}{\sum }p\left(D,D^{u},\theta \right) \end{array}$$

by alternating between performing an* expectation* step and a* maximization* step.

- E-step: given the current estimate of the parameters, calculate the expected value for unobserved latent variables or data.
- M-step: find the parameter that maximizes this quantity. These parameter estimates are then used to determine the distribution of the latent variables in the next E-step.

**Figure 1:**
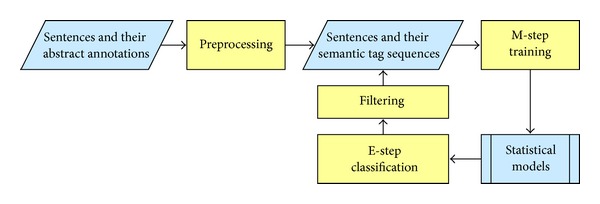
The proposed learning framework of training statistical models from abstract semantic annotations.

We propose a learning framework based on EM to train statistical models from abstract semantic annotations as illustrated in Figure 1. The whole procedure works as follows. Given a set of sentences **S** = {*S*_*i*_, *i* = 1,…, *N*} and their corresponding semantic annotations **A** = {*A*_*i*_, *i* = 1,…, *N*}, each annotation *A*_*i*_ is expanded to the flattened semantic tag sequence *C*_*i*_ at initialization step. Based on the flattened semantic tag sequences, the initial model parameters are estimated. After that, the semantic tag sequence ${\hat{C}}_{i}$ is generated for each sentence using the current model, $\hat{C}=\{{\hat{C}}_{i},i=1,\ldots ,N\}$. Then, $\hat{C}$ is filtered based on a score function which measures the agreement of the generated semantic tag sequences with the actual flattened semantic tag sequences. In the* maximization* step, model parameters are reestimated using the filtered $\hat{C}$. The iteration continues until convergence. The details of each step are discussed in Figure 1.

### 3.1. Preprocessing

Given a sentence labeled with an abstract semantic annotation as shown in Table 1, we first expand the annotation to the flattened semantic tag sequence as in Table 1(a). The provision of abstract annotations implies that the semantics encoded in each sentence need not be provided in expensive token style. Obviously, there are some input words such as articles, which have no specific semantic meanings. In order to cater for these irrelevant input words, a   DUMMY tag is introduced in the preterminal position. Hence, the flattened semantic tag sequence is finally expanded to the semantic tag sequence as in Table 1(b).

### 3.2. Expectation with Constraints

During the* expectation* step, that is, calculating the most likely semantic tag sequence given a sentence, we need to impose the following two constraints which are implied from abstract semantic annotations.

1. Considering the calculated semantic tag sequence as a hidden state sequence, state transitions are only allowed if both current and next states are listed in the semantic annotation defined for the sentence.
2. If a lexical item is attached to a preterminal tag of a flattened semantic tag, the semantic tag must appear bound to that lexical item in the training annotation.

To illustrate how these two constraints are applied, the sentence “I want to return on Thursday to Dallas” with its annotation “RETURN(TOLOC(CITY(Dallas)) ON(DATE(Thursday)))” is taken as an example. The transition from   RETURN+TOLOC+CITY to   RETURN is allowed since both states can be found in the semantic annotation and follows constraint 1. However, the transition from   RETURN to   FLIGHT is not allowed as it does not follow constraint 1 and   FLIGHT is not listed in the semantic annotation. Also, for the lexical item   Dallas in the training sentence, the only valid semantic tag is   RETURN+TOLOC+CITY because to apply constraint 2   Dallas has to be bound with the preterminal tag   CITY.

We further describe how these two constraints can be imposed into two different models, CRFs and HM-SVMs:

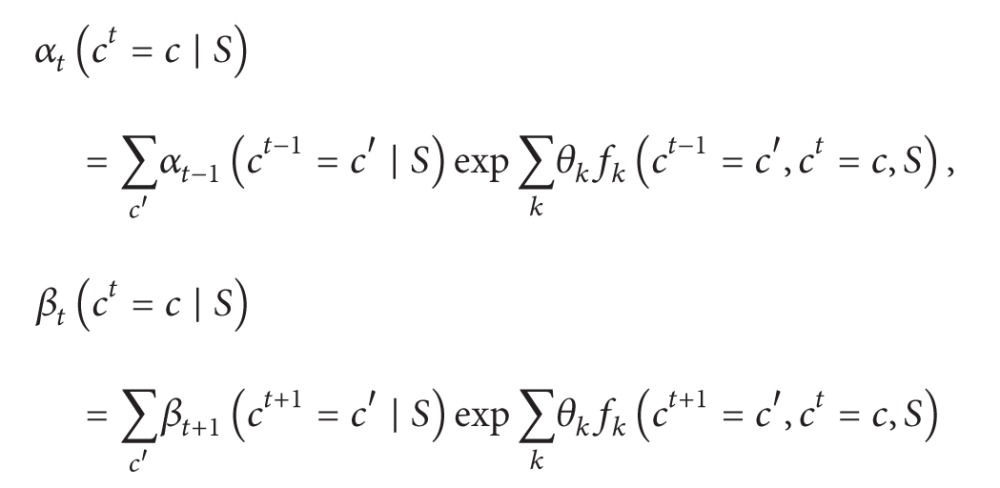
(7)

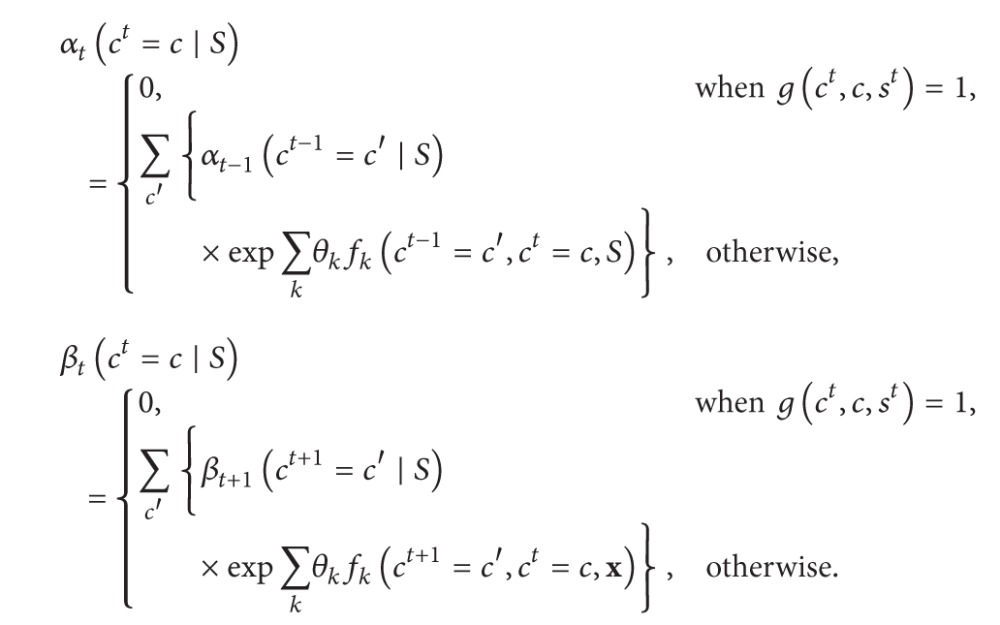
(8)


#### 3.2.1. Expectation in CRFs

The most probable labeling sequence in CRFs can be efficiently calculated using the Viterbi algorithm. Similar to the forward-backward procedure for HMM, the marginal probability of states at each position in the sequence can be computed as

(9) $$\begin{array}{c} P\left(c^{t}=c\;|\;S\right)=\frac{\alpha_{t}\left(c^{t}=c\;|\;S\right)\beta_{t}\left(c^{t}=c\;|\;S\right)}{Z\left(S\right)}, \end{array}$$

where *Z*(*S*) = ∑_*c*_ *α*_*t*_(*c*∣*S*).

The forward values *α*_*t*_(*c* ^*t*^ = *c*∣*S*) and backward values *β*_*t*_(*c* ^*t*^ = *c*∣*S*) are defined in iterative form as (7).

Given the training data *D* = {(*S*_1_, *C*_1_),…, (*S*_*N*_, *C*_*N*_)}, the parameter Θ can be estimated through a maximum likelihood procedure. To calculate the log-likelihood of *L*(Θ) with expectation over the abstract annotation as follows,

(10) L(Θ;Θt)=∑iN‍ ∑CiuP(Ciu ∣ Si;Θt)log⁡P(Ciu ∣ Si;Θ)=∑iN ∑CiuP(Ciu ∣ Si;Θt)∑t ∑kθkfk(c′,c,Si)−∑iklog⁡Z(Si),

where *C*_*i*_^*u*^ is the unknown semantic tag sequence of the *i*th word sequence and *Z*(*S*_*i*_) = ∑_*c*_exp⁡(∑_*t*_∑_*k*_ *θ*_*k*_ *f*_*k*_(*c* ^*t*−1^, *c* ^*t*^, *S*_*i*_)). It can be optimized using the same optimization method as in standard CRFs training.

To infer the word-level semantic tag sequences based on abstract annotations, (7) are modified as shown in (8), where *g*(*c* ^*t*^, *c*, *s* ^*t*^) is defined as follows:

(11) $$\begin{array}{c} g\left(c^{t},c,s^{t}\right)=\max\{\begin{array}{cc} 1, & c\;\;\text{is}\;\;\text{not}\;\;\text{in}\;\;\text{the}\;\;\text{allowable}\\ & \text{semantic}\;\;\text{tag}\;\;\text{list}\;\;\text{of}\;\;S,\\ 1, & c\;\;\text{is}\;\;\text{not}\;\;\text{of}\;\;\text{class}\;\;\text{type}\;\;\text{and}\\ & s^{t}\;\;\text{is}\;\;\text{of}\;\;\text{class}\;\text{type},\\ 0, & \text{otherwise}. \end{array} \end{array}$$

#### 3.2.2. Expectation in HM-SVM

To calculate the most likely semantic tag sequence $\hat{C}$ for each sentence *S*, $\hat{C}={\arg\;\max}_{C\in C}F(S,C)$, we can decompose the discriminant function *F* : *S* × *C* → *R* into two components, *F*(*S*, *C*) = *F*_1_(*S*, *C*) + *F*_2_(*S*, *C*), where

(12) F1(S,C)=∑σ∈c,τ∈cδ(σ,τ)∑l=1T[[cl−1=σ∧cl=τ]],F2(S,C)=∑σ∈c  ∑l=1Tγ(sl,σ)[[cl=σ]].

Here, *δ*(*σ*, *τ*) is considered as the coefficient for the transition from state (or semantic tag) *σ* to state *τ* while *γ*(*s* ^*l*^, *σ*) can be treated as the coefficient for the emission of word *s* ^*l*^ from state *σ*. They are defined as follows:

(13) δ(σ,τ)=∑i,C¯αi(C¯)∑m=1|C¯|[[c¯m−1=σ∧c¯m=τ]],γ(sl,σ)=∑i,m∑C[[cm=σ]]αi(C)k(sl,sim),

where *k*(*s* ^*l*^, *s*_*i*_^*m*^) = 〈Ψ(*s* ^*l*^), Ψ(*s*_*i*_^*m*^)〉 describes the similarity of the input patterns Ψ between word *s* ^*l*^ and word *s*_*i*_^*m*^, the *m*th word in the training example *i*, and *α*_*i*_(*C*) is a set of dual parameters or Lagrange multiplier of the constraint associated with example *i* and semantic tag sequence *C* as in (4). Using the results derived in (13), Viterbi decoding can be performed to generate the best semantic tag sequence.

To incorporate the constraints as defined in the abstract semantic annotations, the values of *δ*(*σ*, *τ*) and *γ*(*s* ^*l*^, *σ*) are modified for each sentence:

(14) δ(σ,τ)={0,when  g(σ,τ)=1,∑i,C¯αi(C¯)∑m[[c¯m−1=σ∧c¯m=τ]],otherwise,γ(sl,σ)={0,when  h(σ,sl)=1,∑i,m ∑C[[cm=σ]]αi(C)k(sl,sim),otherwise,

where *g*(*σ*, *τ*) and *h*(*σ*, *s* ^*l*^) are defined as follows:

(15) g(σ,τ)={1,τ  is  not  in  the  allowable  semantic  tag  list,0,otherwise,h(σ,sl)={1,σ  is  not  of  class  type  and  sl  is  of  class  type,0,otherwise,

where *g*(*σ*, *τ*) and *h*(*σ*, *s* ^*l*^) in fact encode the two constraints implied from abstract annotations.

### 3.3. Filtering

For each sentence, the semantic tag sequences generated in the* expectation* step are further processed based on a measure on the agreement of the semantic tag sequence *T* = {*t*_1_, *t*_2_,…, *t*_*n*_} with its corresponding abstract semantic annotation *A*. The score of *T* is defined as

(16) $$\begin{array}{c} \text{Score}\left(T\right)=2*\frac{S_{\text{recall}}*S_{\text{precision}}}{S_{\text{recall}}+S_{\text{precision}}}, \end{array}$$

where *S*_precision_ = *N*_*r*_/*n*,  *S*_Recall_ = *N*_*r*_/*p*. Here, *N*_*r*_ is the number of the semantic tags in *T* which also occur in *A*, *n* is the number of semantic tags in *T*, and *p* is the number of semantic tags in the flattened semantic tag sequence for *A*. The score is similar to the *F*-measure which is the harmonic mean of precision and recall. It essentially measures the agreement of the generated semantic tag sequence with the abstract semantic annotation. We filter out sentences with their score below certain predefined threshold and the remaining sentences together with their generated semantic tag sequences are fed into the next* maximization* step. In our experiments, we empirically set the threshold to 0.1.

### 3.4. Maximization

Given the filtered training examples from the* filtering* step, the parameters Θ are adjusted using the standard training algorithms.

For CRFs, the parameter Θ can be estimated through a maximum likelihood procedure. The model is traditionally trained by maximizing the conditional log-likelihood of the labeled sequences, which is defined as

(17) $$\begin{array}{c} L\left(\Theta \right)=\overset{N}{\underset{i=1}{\sum }}\log\left(P\left(C_{i}\;|\;S_{i};\Theta \right)\right), \end{array}$$

where *N* is the number of sequences.

The maximization can be achieved gradient ascent where the gradient of the likelihood is

(18) ∂∂θk=∑i=1N ∑tfk(cit−1,cit,Si,t)−∑i=1N ∑Spθ(C ∣ Si)∑tfk(ct−1,ct,Si,t).

For HM-SVMs, the parameters Θ = *w* are adjusted so that the true semantic tag sequence *C*_*i*_ scores higher than all the other tag sequences *C* ∈ *C*_*i*_ : = *C*∖*C*_*i*_ with a large margin. To achieve the goal, the optimization problem as stated in (3) is solved using an online learning approach as described in [4]. In short, it works as follows: a pattern sequence *S*_*i*_ is presented and the optimal semantic tag sequence ${\hat{C}}_{i}=f(S_{i})$ is computed by employing Viterbi decoding. If ${\hat{C}}_{i}$ is correct, no update is performed. Otherwise, the weight vector *w* is updated based on the difference from the true semantic tag sequence $\Delta \Phi =\Phi (S_{i},{\hat{C}}_{i})-\Phi (S_{i},C_{i})$.

## 4. Experimental Results

Experiments have been conducted on the DARPA communicator data (http://www.bltek.com/spoken-dialog-systems/cu-communicator.html/) which were collected in 461 days. From these, 46 days were randomly selected for use as test set data and the remainders were used for training. After cleaning up the data, the training set consists of 12702 utterances while the test set contains 1178 utterances.

The abstract semantic annotations used for training only list a set of valid semantic tags and the dominance relationships between them without considering the actual realized semantic tag sequence or attempting to identify explicit word/concept pairs. Thus, it avoids the need for expensive treebank style annotations. For example, for the sentence “I wanna go from Denver to Orlando Florida on December tenth,” the abstract annotation would be   FROMLOC(CITY) TOLOC(CITY(STATE)) MONTH(DAY).

To evaluate the performance of the model, a reference frame structure was derived for every test set sentence consisting of slot/value pairs. An example of a reference frame is shown in Table 2.

Performance was then measured in terms of *F*-measure on slot/value pairs, which combines the precision (*P*) and recall (*R*) values with equal weight and is defined as *F* = 2∗*P*∗*R*/(*P* + *R*).

We modified the open source of the CRF suite (http://www.chokkan.org/software/crfsuite/) and SVM^HMM^ (http://www.cs.cornell.edu/people/tj/svm_light/svm_hmm.html/) to implement our proposed learning framework. We employed two algorithms to estimate the parameters of CRFs, the stochastic gradient descent (SGD) iterative algorithm [21], and the limited-memory BFGS (L-BFGS) method [22]. For both algorithms, the regularization parameter was empirically set in the following experiments.

### 4.1. Overall Comparison

**Figure 2:**
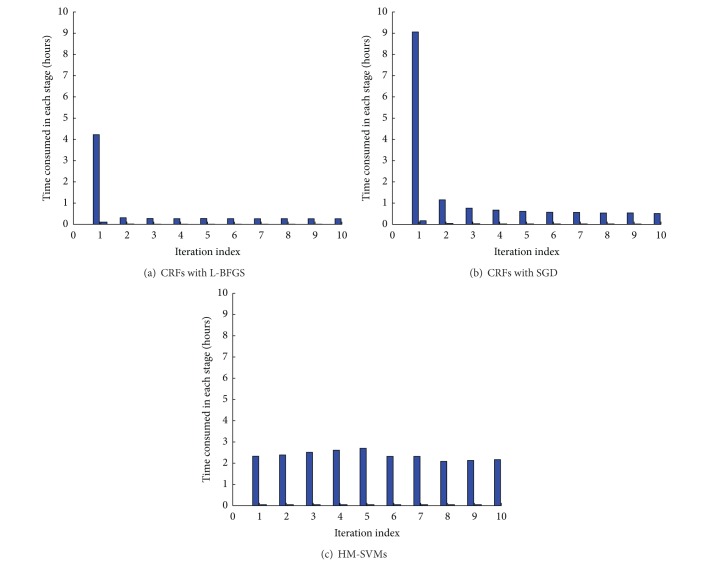
Time consumed in each iteration by CRFs and HM-SVMs.

We first compare the time consumed in each iteration using HM-SVMs or CRFs as shown in Figure 2. The experiments were conducted on the Intel(R) Xeon(TM) model Linux server equipped with 3.00 Ghz processor and 4 GB RAM. It can be observed that, for CRFs, the time consumed in SGD is almost doubled compared to that in L-BFGS in each iteration. However, since SGD converges much faster than L-BFGS, the total time required for training is almost the same. As SGD gives balanced precision and recall values, it should be preferred more than L-BFGS in our proposed learning procedure. On the other hand, as opposed to CRFs which consume much less time after iteration 1, HM-SVMs take almost the same run time for all the iterations. Nevertheless, the total run time until convergence is almost the same for CRFs and HM-SVMs.

**Figure 3:**
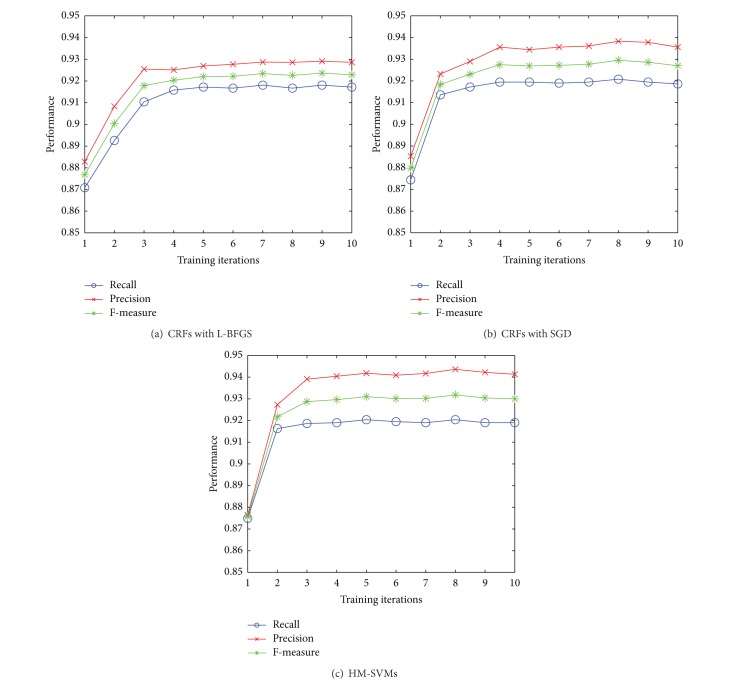
Performance for CRFs and HM-SVMs at each iteration.

Figure 3 shows the performance of our proposed framework for CRFs and HM-SVMs at each iteration. At each word position, the feature set used for both statistical models consists of the current word and the current part-of-speech (POS) tag. It can be observed that both models achieve the best performance at iteration 8 with an *F*-measure of 92.95% and 93.18% being achieved using CRFs and HM-SVMs, respectively.

### 4.2. Results with Varied Features Set

We employed word features (such as current word, previous word, and next word) and POS features (such as current POS tag, previous one, and next one) for training. To explore the impact of the choices of features, we explored with feature sets comprised of words or POS tags occurring before or after the current word within some predefined window size.

**Figure 4:**
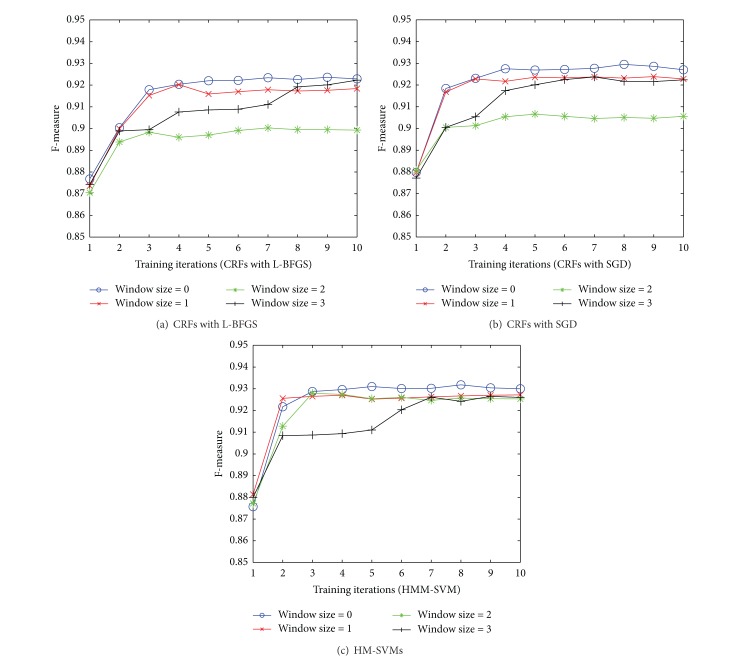
Comparison of performance on models learned with feature sets chosen based on different window sizes.

Figure 4 shows the performance of our proposed approach with the window size varying between 0 and 3. Surprisingly, the model learned with feature set chosen by setting window size 0 gives the best overall performance. Varying window size between 1 and 3 only impacts the convergence rate and does not lead to any performance difference at the end of the learning procedure.

### 4.3. Performance with or without Filtering Step

**Figure 5:**
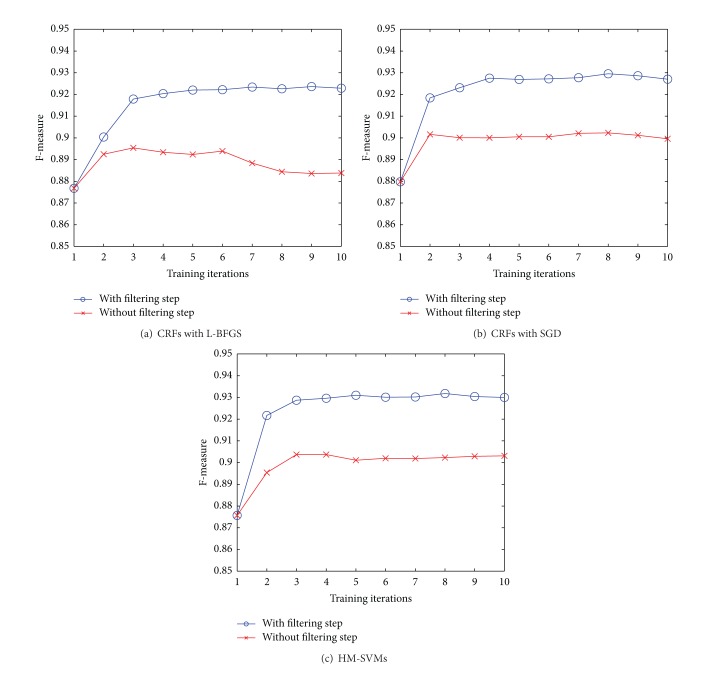
Comparisons of performance with or without the* filtering* stage.

In a second set of experiments, we compare the performance with or without the* filtering* step as discussed in Section 3.3.  Figure 5 shows that the* filtering* step is indeed crucial as it boosted the performance by nearly 4% for CRFs with L-BFGS and 3% for CRFs with SGD and HM-SVMs.

### 4.4. Comparison with Existing Approaches

We compare the performance of CRFs and HM-SVMs with HVS, all trained on abstract semantic annotations. While it is hard to incorporate arbitrary input features into HVS learning, both CRFs and HM-SVMs have the capability of dealing with overlapping features.  Table 3 shows that they outperform HVS with a relative error reduction of 36.6% and 43.3% being achieved, respectively. In addition, the superior performance of HM-SVMs over CRFs shows the advantage of HM-SVMs on learning nonlinear discriminant functions via kernel functions.

We further compare our proposed learning approach with two other methods. One is a hybrid generative/discriminative framework (HF) [23] which combines HVS with HM-SVMs so as to allow the incorporation of arbitrary features as in CRFs. The other is a discriminative approach (DT) based on parse error measure to train the HVS model [24]. The generalized probabilistic descent (GPD) algorithm [25] was employed to adjust the HVS model to achieve the minimum parse error rate.

Table 3 shows that our proposed learning approach outperforms both HF and DT. Training statistical models on abstract annotations allows the calculation of conditional likelihood and hence results in direct optimization of the objective function to reduce the error rate of semantic labeling. On the contrary, the hybrid framework firstly uses the HVS parser to generate full annotations for training HM-SVMs. This process involves the optimization of two different objective functions (one for HVS and another for HM-SVMs). Although DT also uses an objective function which aims to reduce the semantic parsing error rate, it is in fact employed for supervised reranking where the input is the *N*-best parse results generated from the HVS model.

## 5. Conclusions

In this paper, we have proposed an effective learning approach which can train statistical models such CRFs and HM-SVMs without using the expensive treebank style annotation data. Instead, it trains the statistical models from only abstract annotations in a constrained way. Experimental results show that, using the proposed learning approach, both CRFs and HM-SVMs outperform the previously proposed HVS model on the DARPA communicator data. Furthermore, they also show superior performance than the two other methods: one is the hybrid framework (HF) combining both HVS and HM-SVMs, and the other is discriminative training (DT) of the HVS model, with a relative error reduction rate of about 25% and 15% being achieved when compared with HF and DT, respectively.

In future work, we will explore other score functions in* filtering* step to describe the precision of the parsing results. Also, we plan to apply the proposed framework in some other domains such as information extraction and opinion mining.

## Figures and Tables

**Table 1:** Abstract semantic annotation and its flattened semantic tag sequence.

Sentence	I want to return to Dallas on Thursday.
Annotation	R ETURN (TOLOC * * (CITY * * (Dallas) ) ON (DATE * * (Thursday)) )
(a) Flattened semantic tag list:	
RETURN R ETURN + TOLOC RETURN + TOLOC + CITY * * ( Dallas) RETURN + ON RETURN + ON + DATE * * (Thursday)	
(b) Expanded semantic tag list:	
RETURN RETURN + DUMMY RETURN + TOLOC RETURN + TOLOC + DUMMY RETURN + TOLOC + CITY * * (Dallas)	
RETURN + ON RETURN + ON + DUMMY RETURN + ON + DATE * * (Thursday) RETURN + ON + DATE * * (Thursday) + DUMMY	

**Table 2:** 

I wanna travel from Denver to San Diego on March sixth.	
Frame	AIR
Slots	FROMLOC *·* CITY = Denver
	TOLOC *·* CITY = San Diego
	MONTH = March
	DAY = sixth

**Table 3:** Performance comparison between the proposed framework and three other approaches (HF denotes the hybrid framework and DT denotes discriminative training the HVS model.

Measurement	HVS	HF	DT	Proposed framework	
				CRFs	HM-SVMs
Recall (%)	87.81	90.99	91.49	92.08	92.04
Precision (%)	88.13	90.25	91.87	93.83	94.36
*F*-measure (%)	87.97	90.62	91.68	92.95	93.18
